# Taking the Long View: An Evolving, Multi-Dimensional Arc to Advancing Aging Policy and Practice

**DOI:** 10.1093/geroni/igaf122.671

**Published:** 2025-12-31

**Authors:** Edward Miller

**Affiliations:** University of Massachusetts Boston, Boston, Massachusetts, United States

## Abstract

Gerontologists influence aging policy and practice in multiple rich and varied ways during their careers. Dr. Edward Miller will draw on more than 35 years working in the field to illustrate the trajectories (often unplanned) that make a career in gerontology so gratifying. This discussion will be informed by his personal history and educational background, and his professional experience as a researcher and citizen of the profession. Dr. Miller has sought to understand the determinants and effects of federal and state policies affecting older adults, especially those in need of long-term services and supports. His research has applied a range of theories and methodological approaches to elucidating varied dimensions of the aging experience. His efforts include promoting the global dissemination of knowledge in his role as an educator and journal editor. Gerontology is a team sport. Dr. Miller has benefited enormously from the colleagueship of his mentors, students, and collaborators.

